# Supplementary material for: A different perspective for nonphotochemical quenching in plant antenna complexes
Source: Nat Commun. 2021 Dec 9;12:7152. doi: 10.1038/s41467-021-27526-8 (PMC8660843; doi:10.1038/s41467-021-27526-8)
Supplement: Supplementary file 1 — Supplementary Information [file 41467_2021_27526_MOESM1_ESM.pdf]

Supplementary Information: A different perspective for nonphotochemical quenching in plant antenna complexes

Edoardo Cignoni[1] Margherita Lapillo[1] Lorenzo Cupellini[1*]
Silvia Acosta-Gutiérrez[2] Francesco Luigi Gervasio[2,3*]
Benedetta Mennucci[1*]

[1] Dipartimento di Chimica e Chimica Industriale, University of Pisa, via G. Moruzzi 13, 56124, Pisa, Italy
[2] Department of Chemistry, University College London, WC1E 6BT London, UK
[3] School of Pharmaceutical Sciences and ISPSO, University of Geneva, CH-1211 Geneva, Switzerland
* email: lorenzo.cupellini@unipi.it; francesco.gervasio@unige.ch; benedetta.mennucci@unipi.it

Supplementary Methods

Unbiased Molecular Dynamics

We briefly describe the simulation protocol adopted for the $\mathrm{cMD_{CryoEM}}$ and the $\mathrm{cMD_{Open}}$ unbiased MDs. The two simulations differ in the preparation of the starting structure, so we first explain how the starting structure is prepared in the two cases, and then we provide some details on the simulation protocol. Additional information can be found in Ref.[1].

$\mathrm{cMD_{CryoEM}}$

The starting structure for the $\mathrm{cMD_{CryoEM}}$ simulation of CP29 is the cryo-EM structure resolved by Wei et al.[2] (PDB code: 3JCU, chain R). We note that this structure lacks the Chl *b*614, which is instead resolved in the X-ray structure[3] (PDB code: 3PL9). In addition to the pigments (10 Chl *a*, 3 Chl *b*, 1 Lut, 1 Vio, 1 Neo) and one lipid (DPPG) associated with the antenna, seven interstitial water molecules present in the cryo-EM structure were retained, and the N-terminus, lacking its first 11 residues, capped with an ACE group. The protein protonation was determined by the H++[4] web server. The complex has then been inserted in a pre-equilibrated, water-solvated DOPC bilayer membrane (370 DOPC molecules, 25300 waters).

$\mathrm{cMD_{Open}}$

A representative structure from the Open basin was extracted and used as a starting structure for the cMD_{Open} simulations. The representative structure has been determined with a procedure similar to the one presented in Ref.[5]. First, a structure is chosen at random from those found within the Open basin and taken as reference. Then, each other structure is aligned to the reference, and a new representative structure is determined as a weighted average of the aligned structures, using the metadynamics weights to perform the average. The process is repeated until convergence, i.e., until the difference (using the RMSD as a similarity measure) between two successive representative structures is lower than a threshold. Finally, the structure nearest to the converged reference is chosen.

Simulation protocol

The simulation protocol was adapted from the one by Ogata et al.[6]. The system was first minimized in three stages: (i) minimization of the solvent water molecules, (ii) minimization of the water molecules and the bilayer membrane, and (iii) full minimization of the system. Each optimization stage consisted of 5000 steps of steepest descent followed by 5000 steps of conjugate gradient. Next, the system was heated from 0 K to 100 K in a 2 ps long NVT simulation, and from 100 K to 300 K in a 100 ps long NPT simulation, while restraining the membrane-protein-pigment complex with a harmonic potential (force constant of 10 kcal mol^{-1} $Å^{-1}$). Then, the system was relaxed with a 6 ns NPT simulation, gradually releasing the previous constraints. The MD simulations were then extended by 3 μs production for cMD_{CryoEM} and by 2 μs for the two cMD_{Open} replicas. The simulations were performed with AMBER 18[7], with an integration step of 2 fs. Anisotropic periodic boundary conditions were applied. Long-range electrostatics was treated with Particle Mesh Ewald (PME). The Langevin thermostat and the Monte Carlo barostat have been employed to ensure a constant temperature and pressure. SHAKE has been used to place holonomic constraints on bonds involving hydrogen atoms. The protein was described by the AMBER ff14SB[8] force field, the membrane lipids by lipid14[9], and water molecules by TIP3P[10]. Chl *a* parameters were taken from the literature[11], and missing parameters for Chl *b* were taken from GAFF[12]. Carotenoids parameters, instead, have been previously developed in our group[13].

Parallel Tempering in the Well-Tempered Ensemble

The Parallel Tempering[14] simulation in the Well-Tempered Ensemble[15,16] (PT-WTE) was run with 15 replicas, each started from the last, equilibrated frame of the cMD_{CryoEM} simulation and further equilibrated (10 ns) in the NPT ensemble at the corresponding temperature. The temperature for the i-th replica was determined as:

$$T_i = T_0 e^{ki} \tag{1}$$

with T_0 = 300 K (the temperature of the first replica) and k = 0.016. After equilibration, a parallel tempering well-tempered metadynamics simulation was run with the potential energy of the system as CV. Gaussians were deposited every 500 steps with a height of 5 kJ mol^{-1}, a bandwidth of 1000 kJ mol^{-1} and a bias factor of 20. This short metadynamics ensures a sufficient degree of overlap between the potential energy distributions of the replicas, thereby improving the acceptance probability of Monte-Carlo exchanges between replicas at different temperature during the production run. A 1 μs production run is then started for each replica, for a total of 15 μs of aggregate sampling. In order to preserve an optimal overlap between the potential energy distributions of neighboring replicas, a quasi-static regime for the underlying bias potential was necessary, where Gaussians were added rarely (every 50000 steps) during the course of the production simulation. Walls were included in order to prevent the dissociation of chlorophylls and the neoxanthin from the protein when exploring the phase space associated with higher temperatures.

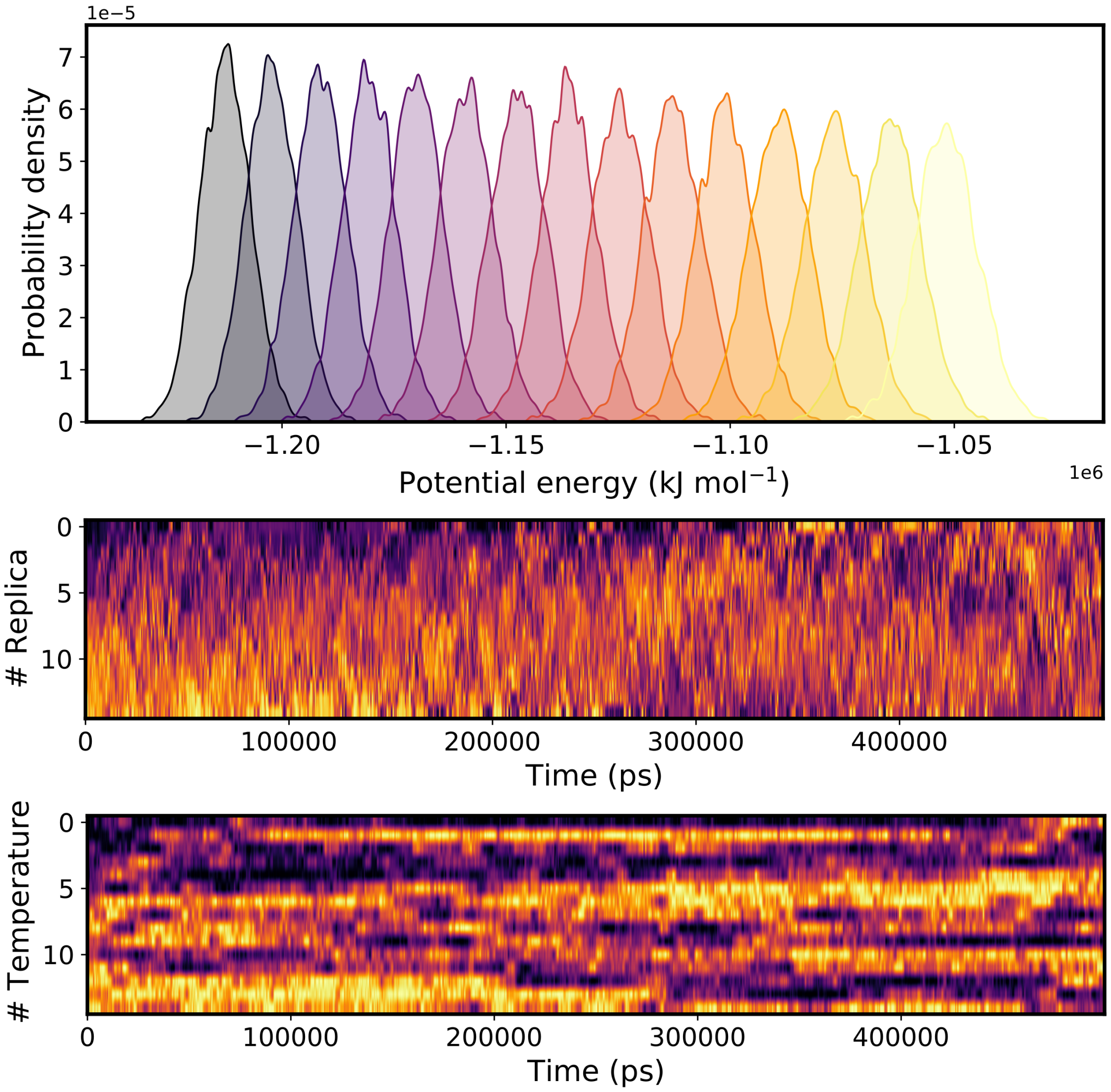

Supplementary Figure 1: Replica exchanges in the PT-WTE Simulation. **a** Probability density estimates for the potential energy distributions of each replica in the PT-WTE simulation. Neighboring replicas do show overlapping potential energy distributions. **b** Replica exchanges along the simulation. **c** Diffusion in temperature space for each replica along the simulation.

Metadynamics

Metadynamics (MetaD) simulations have been performed with GROMACS 2018.6[17] simulation package patched with the PLUMED library[18], version 2.5.1[19]. Conversion between AMBER and GROMACS has been handled with ParmEd[20].

Equations of motion have been integrated using the leap-frog integrator with a 2 fs timestep. Holonomic constraints have been imposed on hydrogen bonds with the LINCS algorithm[21]. A cutoff of 10 Å has been applied for the electrostatic and van der Waals short-range interactions, combined with Particle Mesh Ewald (PME) for the treatment of the long-range electrostatics. The simulations are carried out in the NPT ensemble, with the employment of the velocity-rescaling thermostat from Bussi[22] and the Parrinello-Rahman pressure coupling method[23]. Periodic Bound-

ary Conditions (PBCs) have been applied.

Multiple Walkers Well-Tempered Metadynamics

The preliminar multiple walkers well-tempered metadynamics[24,25] has been run using the $P1_s$ and $P1_l$ angles as collective variables (CVs). In this simulation, Gaussians are deposited with a time interval of 1 ps, a height of 1.0 kJ mol^{-1}, a standard deviation of 0.01 rad and a bias factor of 10. The bias has been stored on a grid to speed up the simulation. Harmonic walls have been applied to the sine of both CVs. Two walkers have been employed to rapidly explore a huge portion of conformational space.

Well-Tempered Metadynamics with Path CVs

An additional well-tempered metadynamics with path CVs[26] has been employed in order to improve on the previous CVs and increase the transitions between the cryo-EM and the Open structures. The path has been constructed from a starting steered MD simulation connecting the two basins, and selecting six landmarks along the MD so as to obtain an average interframe separation of 1.23 Å. The path was then parametrized according to Ref.[26]. We have used the RMSD as a metric to compare the structures from the metadynamics to those of the reference path landmarks. The RMSD is computed by first aligning on the Cα of the residues of helix B,C,A and D, the lutein, and the F211 and W226 residues. Then, the RMSD is computed on the previous selection with the exclusion of helix C. The λ parameter of the path CVs was set to 1.87 Å^{-2}.

Analysis of MD simulations

The analysis of the trajectories, the featurization, dimensionality reduction and clustering were performed with MDTraj[27], PyEMMA[28], Scikit-Learn[29], and in-house Python scripts.

Analysis of the PT-WTE simulation

The PT-WTE simulation was reweighted prior to the analysis by employing the final bias on the potential energy as a static bias, from which the metadynamics weights have been computed. In addition to the bias on the potential energy, we have included the bias due to the walls on both Chl *a*612 and Chl *a*603 in the reweighting. Then, only those frames characterized by a high statistical weight have been employed for the subsequent analysis. In this way, 48.8 % of the frames of the 300 K replica was retained, accounting for the 99.8 % of the total weight of the trajectory. All the distributions reported for the PT-WTE simulation are reweighted ones, i.e., the probability density of an observable ϕ is estimated as:

$$f(\phi) = \frac{\sum_i^N \omega_i K(\phi; \phi_i, h)}{\sum_i^N \omega_i} \tag{2}$$

where $K(\phi; \phi_i, h)$ is a normalized kernel centered in ϕ_i with parameter h, and where ω_i is the statistical weight of the i-th frame. The probability density for non-periodic variables has been estimated using a Gaussian kernel with bandwidth h, whereas the density of periodic variables has been estimated with a von Mises kernel with a concentration parameter h.

dihedral PCA and clustering

The dimensionality reduction analysis was carried out employing residues of the main helices A and B, and residues on the lumenal side of the complex. More in detail, we have selected residues starting from R101 to D123, belonging to helix B and up to the beginning of helix E, and residues from A201 to T236, which belong to helix A up to the end of helix D. The analysis has been

performed using the ϕ, ψ, χ_1 and χ_2 angles of each aminoacid. The cosine and sine of each angle have been employed as features for the following dimensionality reduction step, for a total of 386 features. Dimensionality reduction has been carried out with PCA.

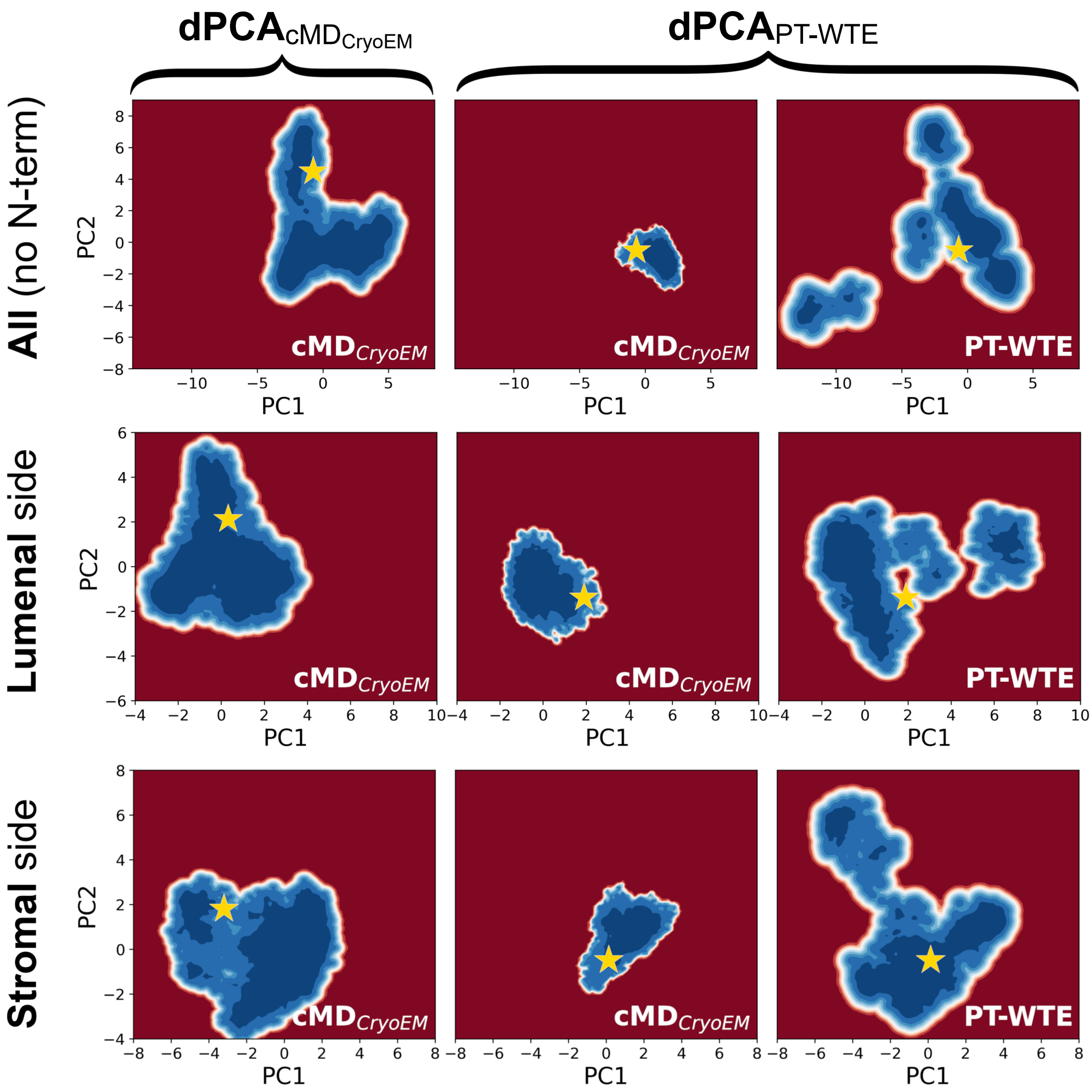

Supplementary Figure 2: Conformational sampling in the PT-WTE and in the cMD$_{\text{CryoEM}}$. The sampling is visualized in a dPCA reduced space. The first column displays the dPCA space determined from the cMD$_{\text{CryoEM}}$ simulation (dPCA$_{cMD_{\text{CryoEM}}}$), while the second and third columns display the dPCA space determined from the PT-WTE simulation (dPCA$_{PT-WTE}$). Each row indicates the portion of protein included in the dPCA analysis. The inset shows what simulation is projected in the dPCA space. The yellow star indicates the position of the cryo-EM structure (PDB: 3JCU). In all cases, the conformational space sampled in the PT-WTE simulation is larger than the one sampled in the cMD$_{\text{CryoEM}}$ simulation.

The clustering was performed employing the first 30 principal components of the dPCA latent space. We have used an agglomerative clustering algorithm, employing the Ward's method for performing each merge. The best number of clusters was explored ranging from a minimum of

two clusters up to a maximum of nine clusters, and monitoring the Bayesian Information Criterion (BIC), the Calinsky-Harabasz, and the mean silhouette scores. A final number of six clusters was determined from inflections in the scores curves. The final clustering and the clustering scores are shown in Figure 3.

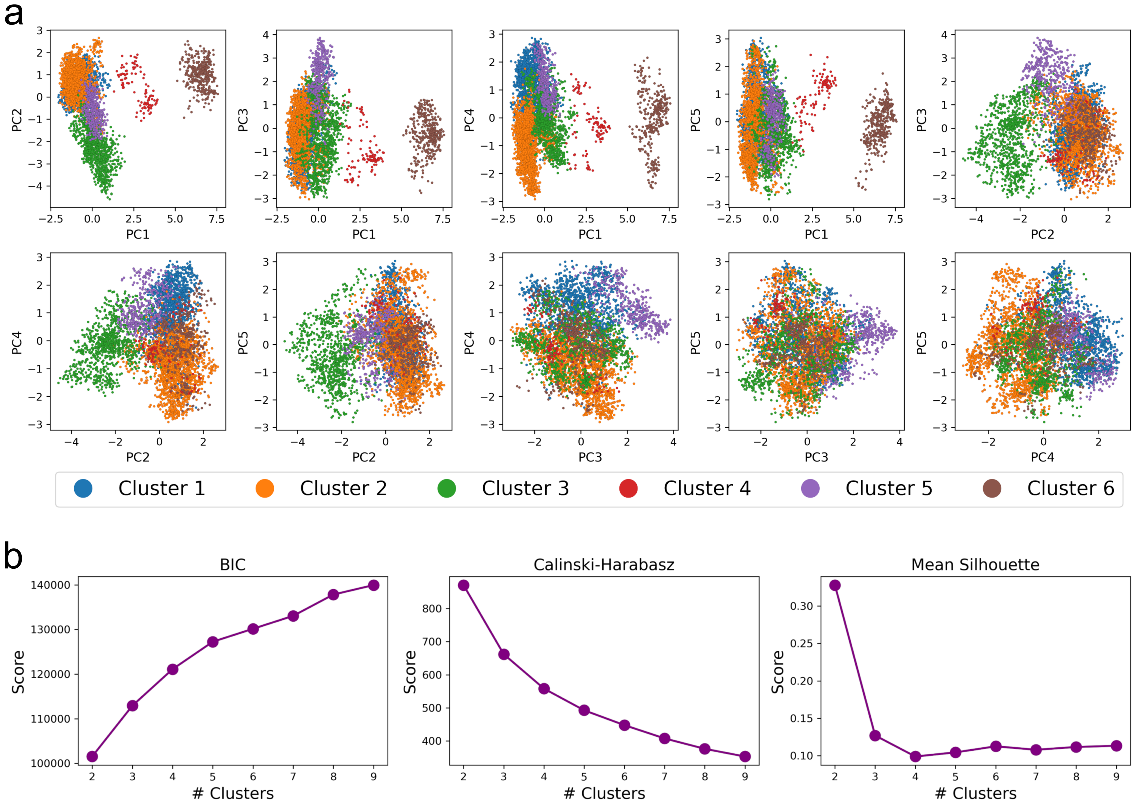

Supplementary Figure 3: Clusters identified in the PT-WTE simulation. **a** dPCA space colored according to the cluster label, for various combinations of the principal components. The first principal component separates well the red and brown clusters from the others. The blue, orange, green, and purple clusters are resolved only when considering principal components corresponding to smaller eigenvalues. **b** Scores monitored to select the optimal number of clusters.

Electronic analysis of quenching

A commonly accepted mechanism of chlorophyll quenching involves the excitation energy transfer (EET) from the Chl's lowest singlet excited state Q_y and the dark S_1 state of the neighboring carotenoid, followed by rapid decay to the ground state with dissipation of the excitation energy as harmless heat. Following what is commonly done in the literature,[30–32], we have modeled EET in the Förster weak coupling limit by assuming a constant spectral overlap between the Chl Q_y emission and the Car S_1 absorption spectra. In this approximation, the LHC can tune the EET quenching through the modulation of the EET coupling V_{EET}. The EET coupling can be written as a sum of a long-range Coulomb contribution and a short-range contribution:

$$V_{\mathrm{EET}} = V_{\mathrm{Coul}} + V_{\mathrm{short}} \quad (3)$$

Estimation of the Coulomb coupling

The Coulomb contribution to the total coupling is approximated as the Coulomb interaction between transition charges (TrEsp method[33]) of the two interacting pigments:

$$V_{\text{Coul}}^{\text{TrEsp}} = \sum_{\substack{i \in \text{Chl} \\ j \in \text{Car}}} \frac{q_i \, q_j}{R_{ij}} \tag{4}$$

The transition charges for the Q_y state of Chls were computed at the TDDFT B3LYP/6-31+G(d) level of theory; those for the S_1 state of Cars were obtained at the DFT-MRCI level of theory.[34] The transition charges are rescaled by a factor of 3.7, analogously to what has been done in Ref[1], in order to be comparable with previous work.[1,31]

Estimation of short-range effects

The short-range contribution V_{short} was estimated for the S_2/Q_y coupling, as the S_2 state is accessible to standard excited-state calculations. V_{short} was computed as the difference between the total coupling V_{EET} and the Coulomb contribution V_{Coul}. The total coupling V_{EET} was obtained from an excited-state calculation of the Car-Chl pair at the TDA/ωB97X-D/6-31+G(d) level of theory followed by the application of the multi-FED-FCD diabatization scheme[35]. This scheme yields the energies of locally-excited (LE) and charge-transfer (CT) states, as well as all couplings between them, includig the EET coupling. Due to the double-excitation nature of the Car S_1, these calculations cannot provide information about this state. The Coulomb coupling V_{Coul} is obtained via direct integration of the transition densities of the two pigments[36]. These calculations were performed on 100 snapshots extracted from clusters 4,5, and 6 (See below). Short-range effects can be estimated geometrically by resorting to an approximation of the electronic wavefunction overlap in terms of the volume of the intersection between the van der Waals regions of the interacting pigments[37]. These van der Waals regions are defined as the union of interlocking spheres positioned on the conjugated atoms of the two pigments. In order to directly compare with our previous work, we use the same radii (1.4 times the van der Waals radius) defined there. This definition of "geometrical overlap" was sufficient to explain the variability of triplet energy transfer (TET) couplings among different Car-Chl pairs. The couplings involved in TET have vanishing Coulomb contribution due to the spin-forbidden nature of singlet-triplet transitions,[38] and only contain short-range terms. We leverage the relationship between overlap and TET couplings to estimate the variability of the short-range contribution. The TET couplings can be as large as 20 cm^{-1} when the overlap is above 20 Å^3, and exponentially drop to $\lesssim$3 cm^{-1} for overlap below 10 Å^3.[37].

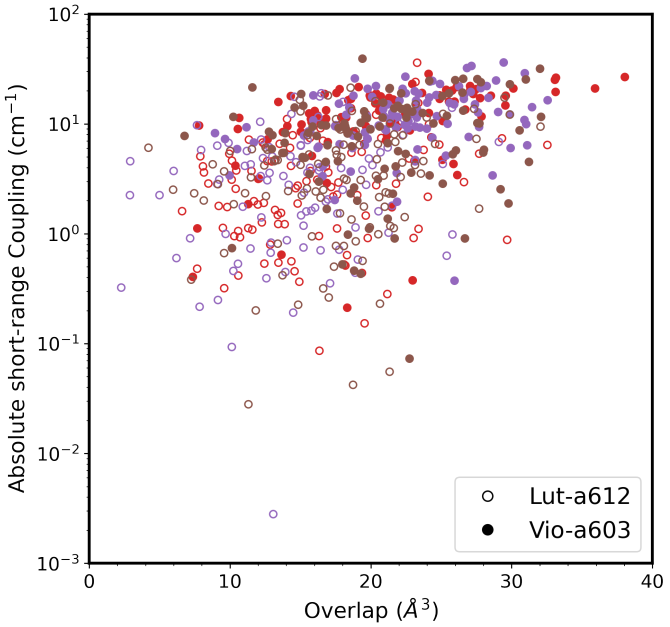

Supplementary Figure 4: Short-range coupling dependence on the overlap. Each point is colored according to the corresponding cluster (red for cluster 4, purple for cluster 5, brown for cluster 6). Empty circles denote the Lut-a612 pair, while filled circles denote the Vio-a603 pair.

As shown in Figure 4, the dependence on the geometrical overlap of the short-range singlet EET S_2/Q_y is very similar to the case of TET[37]. In addition, we note that the order of magnitude of the short-range couplings is comparable to that of Coulomb couplings, indicating that these short-range contributions might have a significant impact on the total EET coupling.

Determination of the Charge-Transfer states and Marcus analysis

The energy of the CT (Car^+Chl^-) state and its coupling with the Q_y state of the carotenoid's central chlorophyll were computed with the same protocol employed in Ref.[39], in order to obtain comparable results. The snapshots employed were extracted from the clusters 4, 5, and 6, by first filtering out structures with a low statistical weight and then selecting points according to the Farthest Point Sampling (FPS) algorithm[40], so as to obtain a set of geometries representative of each cluster. These snapshots (100 per cluster) were employed for performing QM calculations on both Lut-a612 and Vio-a603 pairs, for a total of 300 calculations per Car-Chl pair. The phytyl tail of the chlorophyll was cut after the first aliphatic carbon and kept within the MM region. The excited states are computed at the TDA/ωB97X-D/6-31+G(d) level of theory. The effect of the environment was included through MMPol. Our multi-FED-FCD diabatization scheme[35] has been employed in order to extract the CT energies and couplings. In order to compare with the results of Ref.[39], where the energy of the Chl Q_y state was computed at TD-DFT/ωB97X-D/6-31+G(d) level of theory, we have subtracted from the Q_y energy determined via the diabatization scheme the difference in mean with the Q_y energy obtained in LHCII. Excited-state calculations were performed with a locally modified version of the Gaussian package[41].

The parameters employed for the determination of the charge-separation rates within the framework of Marcus theory were derived following Ref.[39]. In particular, the adiabatic energy of each state was computed as $\Delta G_X = \langle E_X \rangle - \frac{\sigma_X^2}{2k_BT}$, with $X = \text{Chl*}/\text{Car}^+\text{Chl}^-$. The reorganization energy λ for the Car^+Chl^-/Chl* transition was determined from the variance of the energy difference ΔE of the two states as $\frac{\sigma_{\Delta E}^2}{2k_BT}$.

Parameter	Lut-a612 (L1)	Vio-a603 (L2)
E(Chl*) Q_y	15,533 ± 55	15,603 ± 54
E(Car*) S_2	20,853 ± 134	21,251 ± 147
E(Car^+Chl^-)	22,474 ± 183	23,248 ± 194
V(Chl*, Car^+Chl^-)	106 ± 10	183 ± 15
$\lambda_{\text{CT-LE}}$	5935	6526
$\Delta G_{\text{CT-LE}}$	1045	1328

Supplementary Table 1: Energies and couplings in the Car-Chl dimers. The table mirrors Table 1 of Ref.[39]. The average values of each parameter is reported, along with the 95% confidence interval estimated by bootstrap on 10,000 bootstrap samples. Coupling averages are reported as root mean square (RMS). All values are in cm^{-1}. The time required for charge separation is always > 5 ns and is not reported.

Carotenoid geometry optimization

A subset of frames from clusters 4, 5, and 6 was selected for the optimizations. Carotenoid geometries were optimized at the DFT/B3LYP/6-31G(d) level of theory within a QM/MM ONIOM scheme. The QM part consisted of the carotenoid molecule (Lut or Vio) only. The MM part was described with the same force field parameters used in the molecular dynamics. MM Residues within 6 Å from the carotenoid atoms were allowed to move during the optimization.

Semiempirical calculation of the S_1 energies

The carotenoid S_1 energy has been computed with semiempirical CI (SECI), following Ref.[42], where the SECI calculations have proven accurate in predicting the excitation energy changes with geometry. Orbitals for the SECI calculations are determined through a SCF calculation on an open-shell singlet state with two singly occupied orbitals. The SCF calculation is based on the OM2 Hamiltonian[43,44]. The active space comprises all the π and π^* orbitals of the conjugated main chain, selected according to the procedure proposed in Ref.[45]. All single excitations from two reference determinants, closed-shell HF singlet and the doubly excited $\text{HOMO}^2 \rightarrow \text{LUMO}^2$, were included in the multireference CI wavefunction.

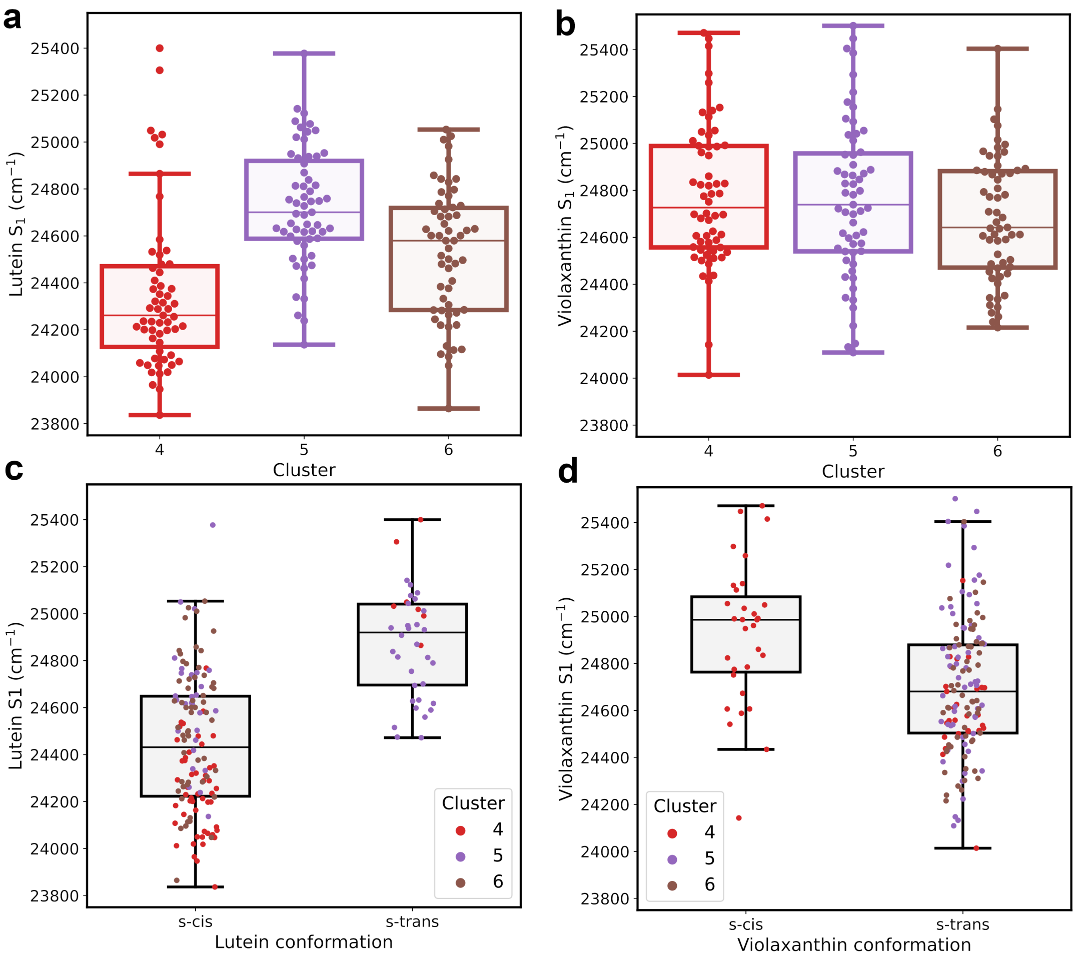

Supplementary Figure 5: Carotenoid S_1 energy. The S_1 energy in each cluster is reported in **a** for Lutein and in **b** for Violaxanthin. The dependence of the S_1 energy on the carotenoid conformation is reported in **c** for Lutein and **d** for Violaxanthin. Each box plot shows the median (horizontal line), the first and third quartile (extension of the box), and the whiskers extend up to 1.5 IQR below and above the first and third quartile. The points from which each box plot is made are shown for clarity. For Lutein (**a** and **c**), n=59, 59, and 58 points from independent quantum chemical calculations are shown for cluster 4, 5, and 6. For Violaxanthin (**b** and **d**), n=58, 59, and 59 points from independent quantum chemical calculations are shown for cluster 4, 5, and 6, respectively.

Supplementary Figures

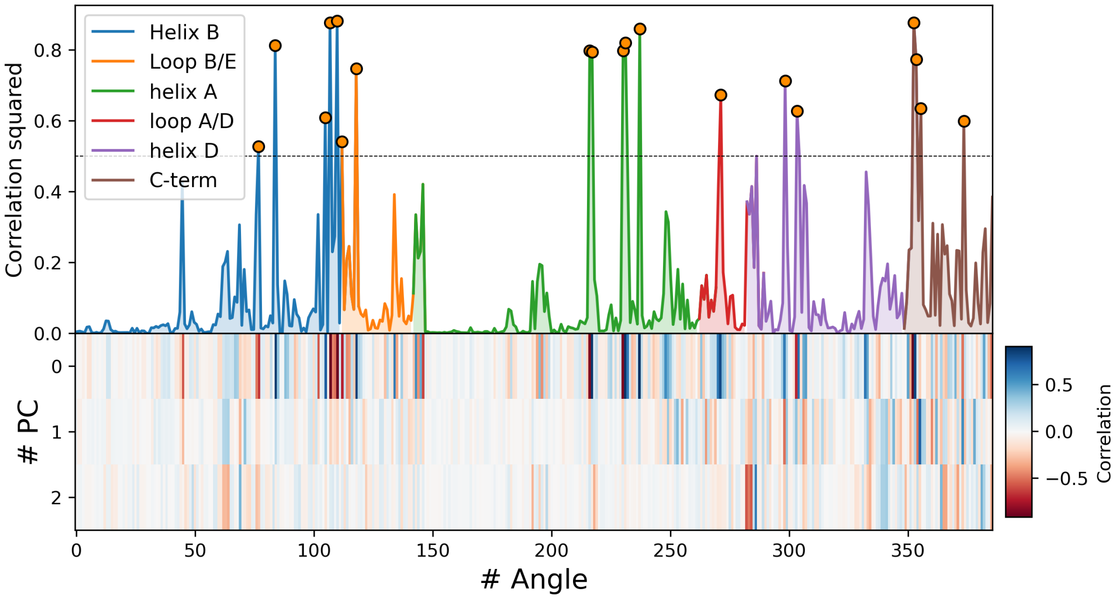

Supplementary Figure 6: Selection of the important residues in the PT-WTE simulation. The panel on the bottom shows the correlation between the aminoacidic angle used for the dPCA analysis and the first three principal components. The top panel shows the square of the correlation for each angle summed over the first three principal components. Each portion of the protein is colored differently. The threshold used to differentiate between more and less important residues is shown as the horizontal dashed line in the upper panel. The angles that strongly correlate with the first three principal components are indicated with an orange circle.

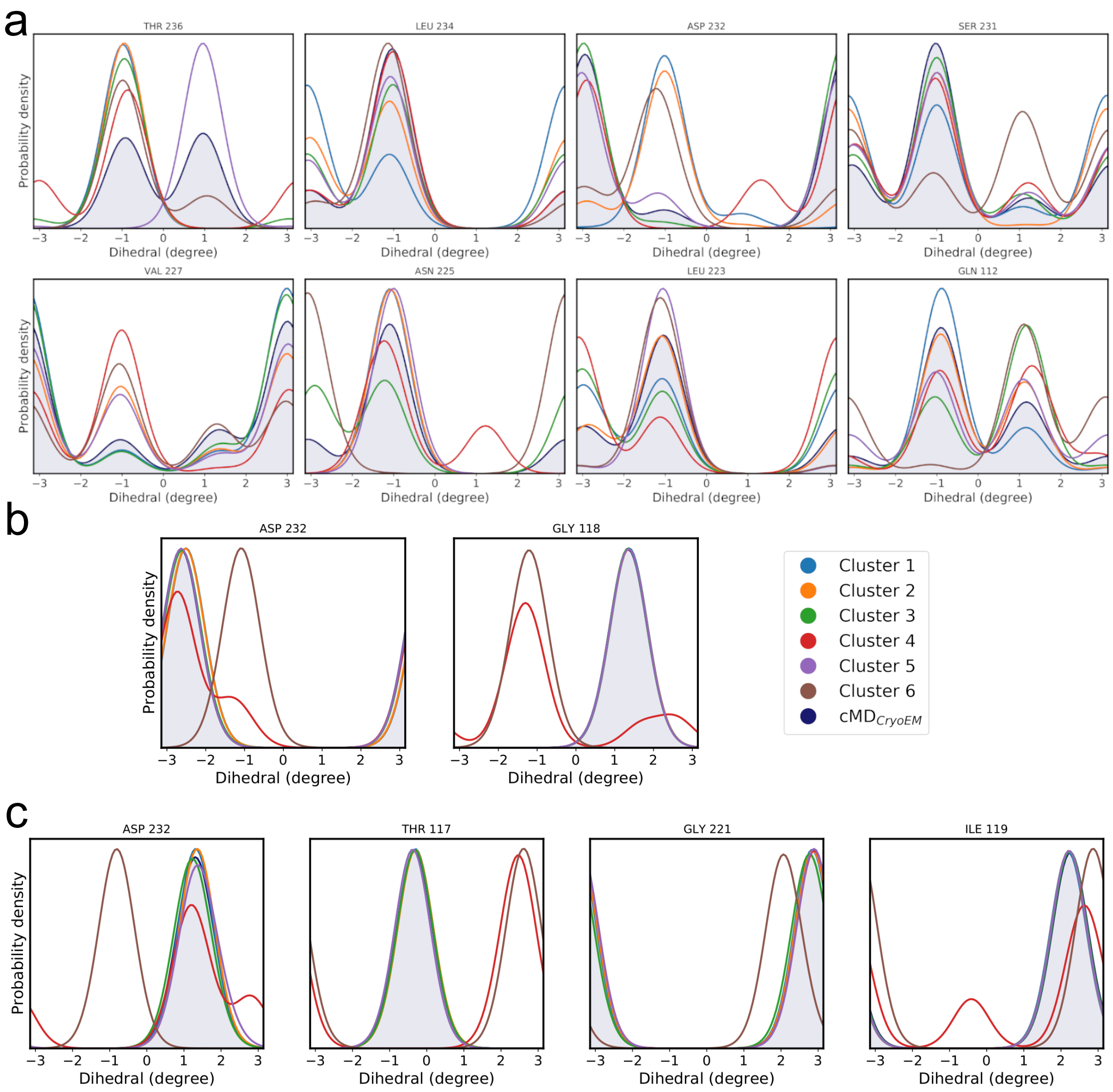

Supplementary Figure 7: Backbone and sidechain angles distributions. Distribution of the backbone (Phi, Psi) and sidechain (Chi1) angles in the clusters identified in the PT-WTE simulation and for the cMD$_{\text{CryoEM}}$ simulation. The area under the distribution of the cMD$_{\text{CryoEM}}$ simulation is shaded for a better visualization. **a** Distributions of the sidechain (Chi1) angles, showing that all of the clusters can be resolved in terms of their sidechain conformations. **b**, **c** Distributions of the backbone (Psi, Phi) angles. Only the clusters brown and red can be distinguished from the rest, showing that they are the only clusters differing in their backbone conformations.

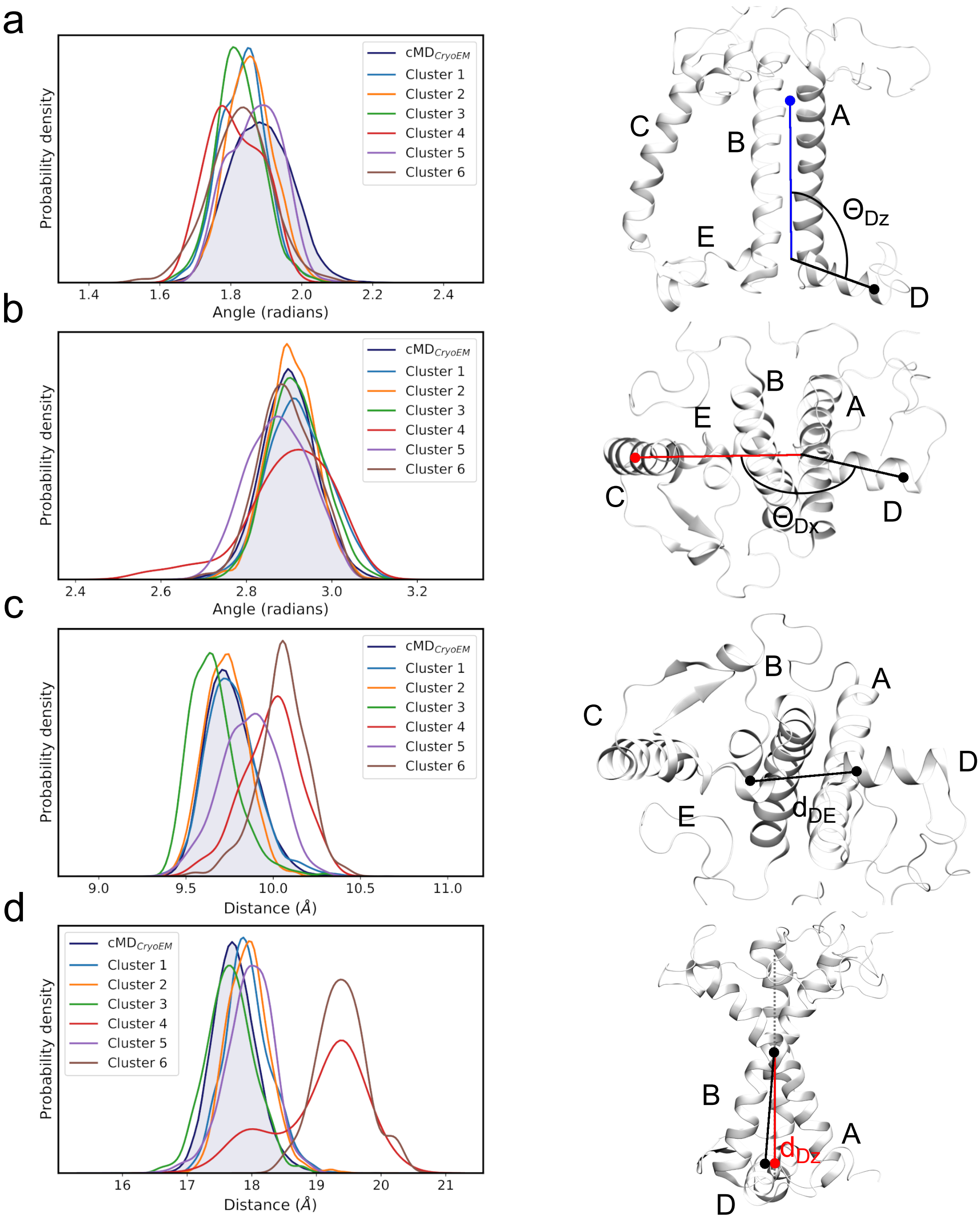

Supplementary Figure 8: Helix D conformations in the PT-WTE simulation. **a** Distributions of the angle θ_{Dz} made by a vector along the helix D and the z axis defined internally to CP29. **b** Distributions of the angle θ_{Dx} made by a vector along the helix D and the x axis, defined internally to CP29. **c** Distributions of the distance d_{DE} between the end of helix E and the beginning of helix D. **d** Distributions of the distance d_{Dz} between the beginning of helix D and the center of helix A, projected along the z axis.

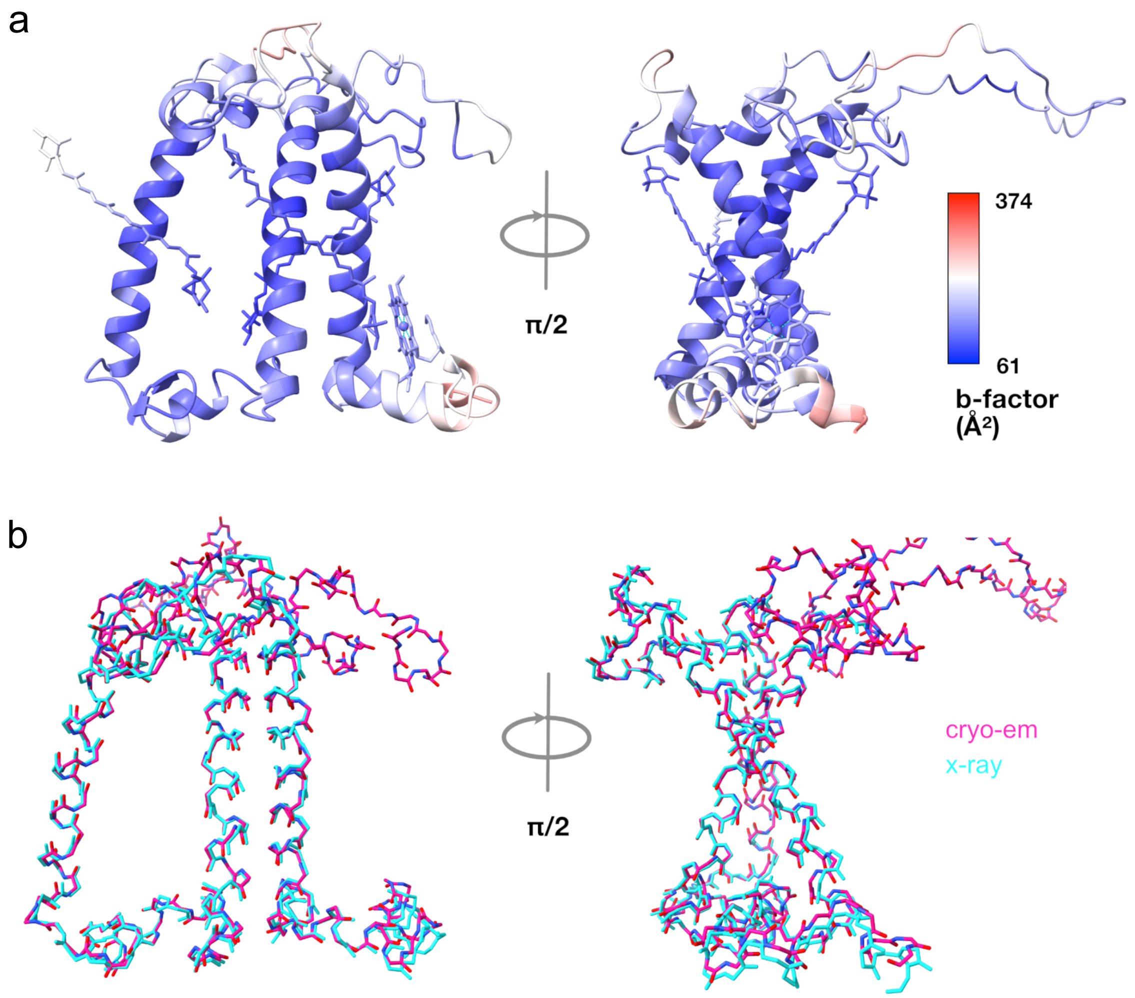

Supplementary Figure 9: Comparison of the cryo-EM and X-ray CP29 structures.. **a** CP29 cryo-EM (PDB: 3JCU) structure colored according to the B-factor. **b** Comparison of the CP29 cryo-EM structure and the CP29 X-ray structure (PDB: 3PL9), showing nearly perfect agreement between the two.

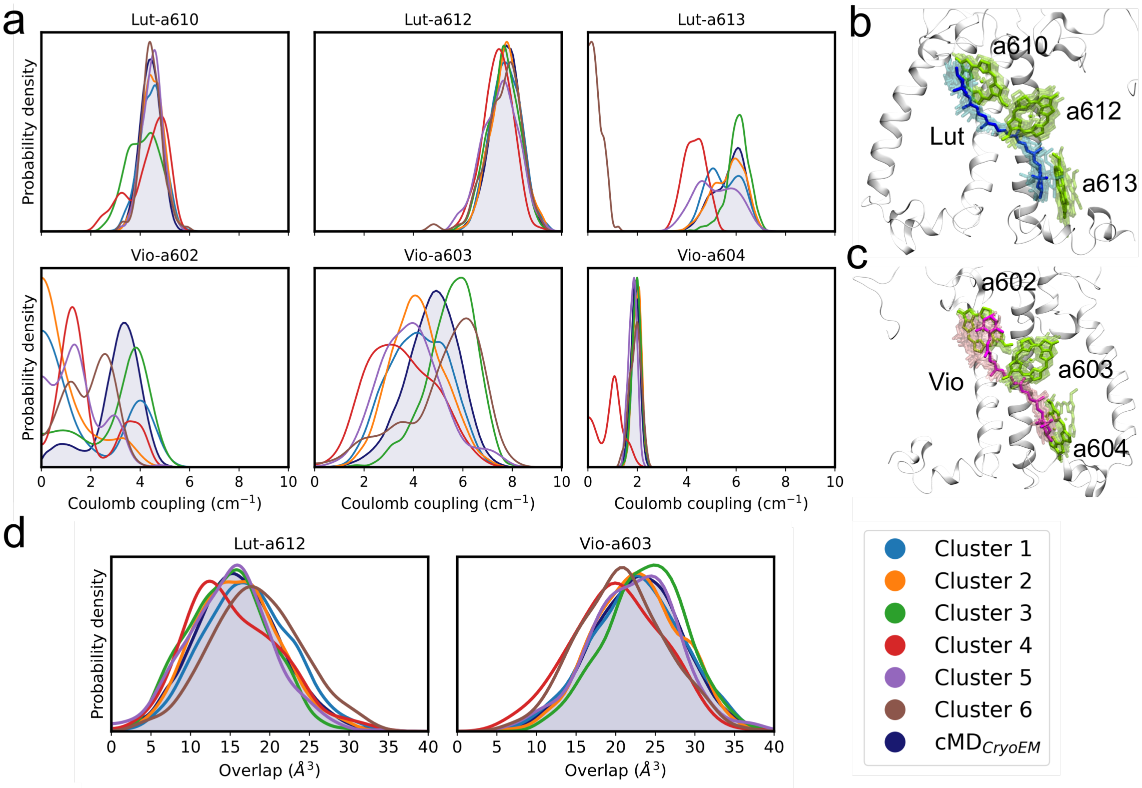

Supplementary Figure 10: Coulomb coupling and overlap within CP29. The distributions are shown for all the clusters of the PT-WTE simulation, and for the cMD$_{\text{CryoEM}}$ simulation. The distribution of the cMD$_{\text{CryoEM}}$ simulation is shaded for an easier visualization. The density domain is bound to positive values only. **a** Distributions of the absolute value of the Coulomb coupling between Lut and Chl *a*610, *a*612, *a*613 (L1) and between Vio and Chl *a*603, *a*603, *a*604 (L2). **b**, **c** fluctuations of the Car-Chl pairs shown in **a**, for the L1 site **b** and the L2 site **c**. **d** Distributions of the overlaps for the pair Lut-*a*612 (L1) and Vio-*a*603 (L2).

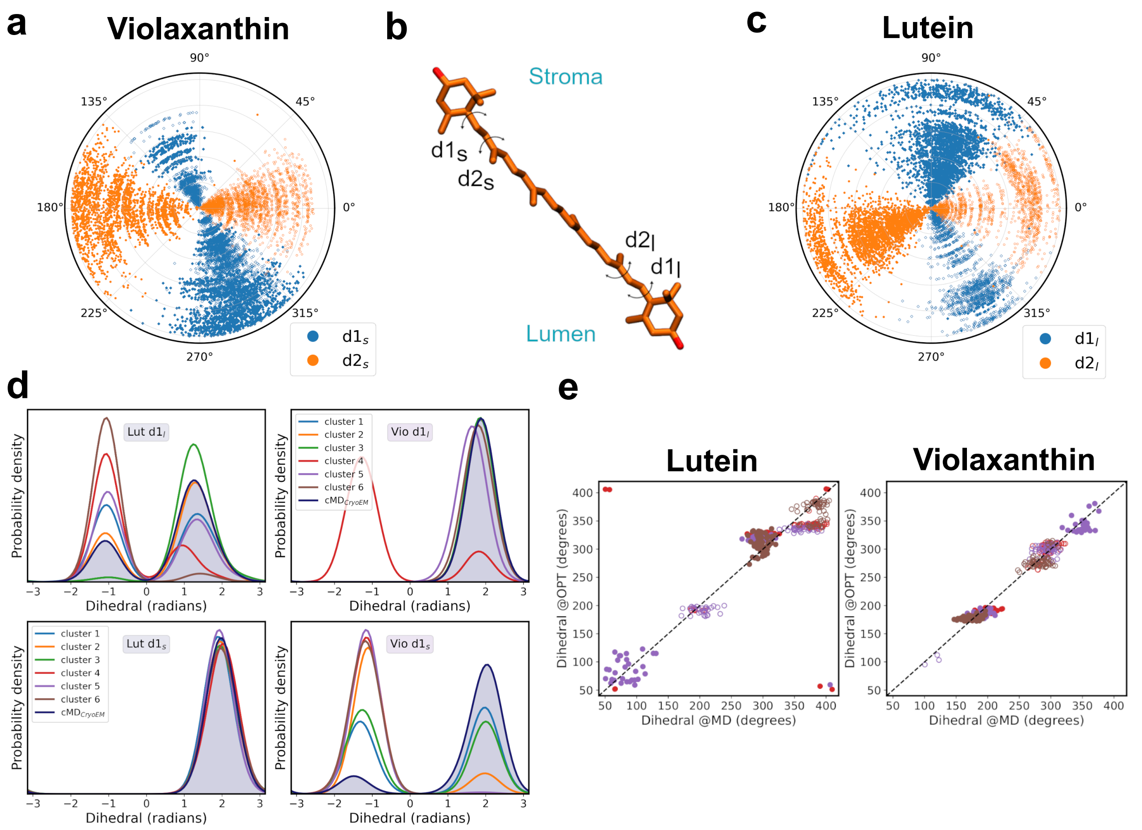

Supplementary Figure 11: Carotenoid conformations in CP29. Dihedral angles of **a** Violaxanthin (stromal side) and **c** Lutein (lumenal side) are shown. Dihedrals corresponding to s-cis conformations are shown as empty circles. The radial coordinate corresponds to the time axis. **b** Definition of the dihedral angles shown in **a**, **c**. **d** Distributions of the first lumenal ($d1_l$) and stromal ($d1_s$) dihedral for both Lutein and Violaxanthin in each cluster. **e** Correlation of the dihedral angles ($d1_l$ and $d2_l$ for Lut, $d1_s$ and $d2_s$ for Vio) before (@MD) and after (@OPT) the carotenoid QM/MM optimization). The first dihedral of the conjugated chain, $d1_X$, $X = s, l$, is represented by filled circles, while the second dihedral $d2_X$, $X = s, l$, is represented by empty circles. Points are colored according to the respective cluster identity (red for cluster 4, purple for cluster 5, brown for cluster 6).

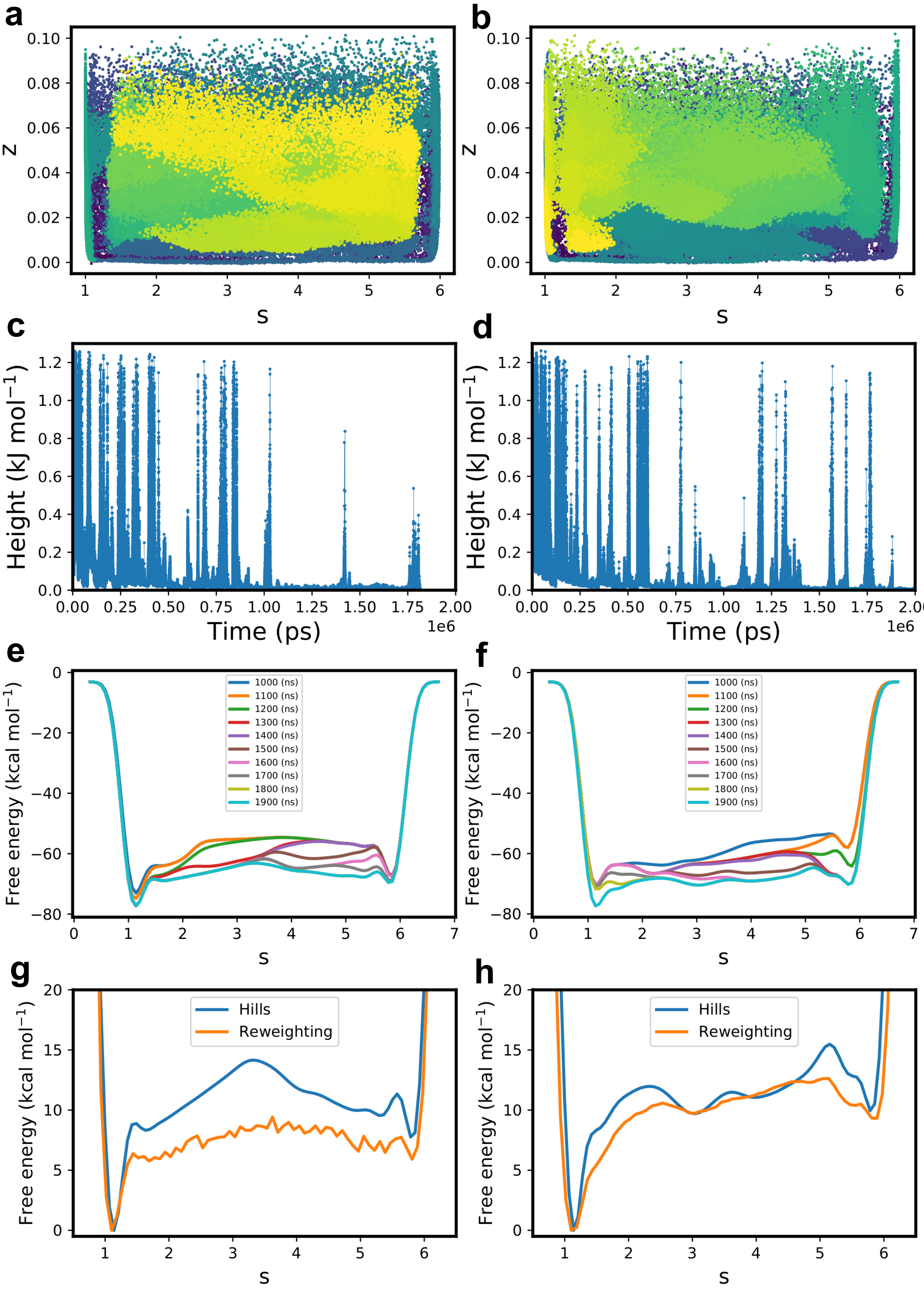

Supplementary Figure 12: Convergence of the path metadynamics. Figures on the left column correspond to the path metadynamics started from the cMD_{CryoEM} simulation **a**, **c**, **e**, **g**, while figures on the right correspond to the path meatadynamics started from the cMD_{Open} simulation **b**, **d**, **f**, **h**. **a**, **b** Exploration of the path CV space. **c**, **d** Heights of the Gaussians deposited along the path metadynamics simulations. **e**, **f** Free energy profile along s every 100 ns starting from 1 μs. **g**, **h** Comparison of the free energy profiles along s computed from reweighting the trajectory and from the addition of the deposited Gaussians.

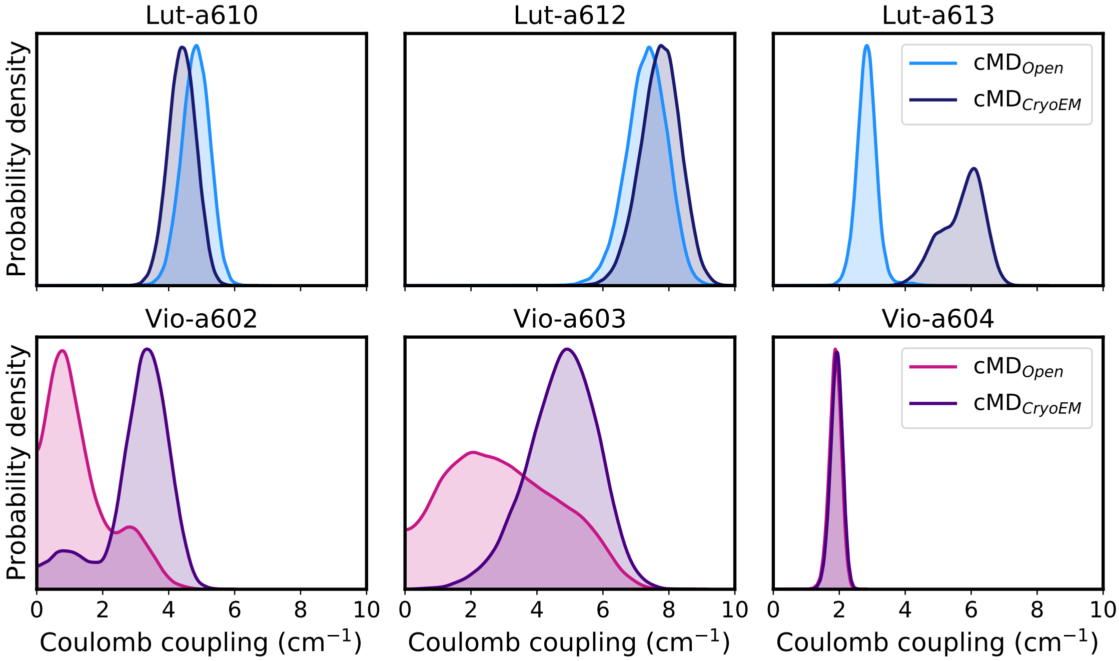

Supplementary Figure 13: Coulomb coupling distributions. Distributions are shown for the cMD_{Open} and the cMD_{CryoEM} simulation, for the three Car-Chl pairs in L1 and L2 sites.

Supplementary References

[1] Lapillo, M., Cignoni, E., Cupellini, L. & Mennucci, B. The energy transfer model of nonphotochemical quenching: Lessons from the minor CP29 antenna complex of plants. *Biochimica et Biophysica Acta (BBA) - Bioenergetics* **1861**, 148282 (2020).

[2] Wei, X. *et al.* Structure of spinach photosystem II–LHCII supercomplex at 3.2–AA resolution. *Nature* **534**, 69–74 (2016).

[3] Pan, X. *et al.* Structural insights into energy regulation of light-harvesting complex cp29 from spinach. *Nature Struct. Mol. Biol.* **18**, 309–15 (2011).

[4] Anandakrishnan, R., Aguilar, B. & Onufriev, A. V. H++ 3.0: Automating pK prediction and the preparation of biomolecular structures for atomistic molecular modeling and simulations. *Nucleic Acids Research* **40**, 537–541 (2012).

[5] Theobald, D. L. & Wuttke, D. S. Accurate structural correlations from maximum likelihood superpositions. *PLoS Computational Biology* **4** (2008).

[6] Ogata, K., Yuki, T., Hatakeyama, M., Uchida, W. & Nakamura, S. All-atom molecular dynamics simulation of photosystem II embedded in thylakoid membrane. *Journal of the American Chemical Society* **135**, 15670–15673 (2013).

[7] Case, D. *et al.* Amber 2018 (2018).

[8] Maier, J. A. *et al.* ff14SB: Improving the Accuracy of Protein Side Chain and Backbone Parameters from ff99SB. *Journal of Chemical Theory and Computation* **11**, 3696–3713 (2015).

[9] Dickson, C. J. *et al.* Lipid14: The amber lipid force field. *Journal of Chemical Theory and Computation* **10**, 865–879 (2014).

[10] Mark, P. & Nilsson, L. Structure and dynamics of the TIP3P, SPC, and SPC/E water models at 298 K. *Journal of Physical Chemistry A* **105**, 9954–9960 (2001).

[11] Zhang, L., Silva, D.-A., Yan, Y. & Huang, X. Force field development for cofactors in the photosystem ii. *Journal of Computational Chemistry* **33**, 1969–1980 (2012).

[12] Wang, J., Wolf, R. M., Caldwell, J. W., Kollman, P. A. & Case, D. A. Development and testing of a general amber force field. *Journal of Computational Chemistry* **25**, 1157–1174 (2004).

[13] Prandi, I. G., Viani, L., Andreussi, O. & Mennucci, B. Combining classical molecular dynamics and quantum mechanical methods for the description of electronic excitations: The case of carotenoids. *Journal of Computational Chemistry* **37**, 981–991 (2016).

[14] Sugita, Y. & Okamoto, Y. Replica-exchange molecular dynamics method for protein folding. *Chemical Physics Letters* **314**, 141–151 (1999).

[15] Bonomi, M. & Parrinello, M. Enhanced sampling in the well-tempered ensemble. *Phys. Rev. Lett.* **104** (2010).

[16] Deighan, M., Bonomi, M. & Pfaendtner, J. Efficient simulation of explicitly solvated proteins in the well-tempered ensemble. *J. Chem. Theory Comput.* **8**, 2189–2192 (2012).

[17] Abraham, M. J. *et al.* Gromacs: High performance molecular simulations through multi-level parallelism from laptops to supercomputers. *SoftwareX* **1-2**, 19–25 (2015).

[18] The PLUMED consortium. Promoting transparency and reproducibility in enhanced molecular simulations. *Nature Methods* **16**, 667–673 (2019).

[19] Tribello, G. A., Bonomi, M., Branduardi, D., Camilloni, C. & Bussi, G. PLUMED 2: New feathers for an old bird. *Computer Physics Communications* **185**, 604–613 (2014).

[20] Shirts, M. R. *et al.* Lessons learned from comparing molecular dynamics engines on the SAMPL5 dataset. *Journal of Computer-Aided Molecular Design* **31**, 147–161 (2017).

[21] Hess, B., Bekker, H., Berendsen, H. J. C. & Fraaije, J. G. E. M. LINCS : A Linear Constraint Solver for Molecular Simulations. *J. Comput. Chem.* **18**, 1463–1472 (1997).

[22] Bussi, G., Donadio, D. & Parrinello, M. Canonical sampling through velocity rescaling. *Journal of Chemical Physics* **126** (2007).

[23] Parrinello, M. & Rahman, A. Polymorphic transitions in single crystals: A new molecular dynamics method. *Journal of Applied Physics* **52**, 7182–7190 (1981).

[24] Raiteri, P., Laio, A., Gervasio, F. L., Micheletti, C. & Parrinello, M. Efficient reconstruction of complex free energy landscapes by multiple walkers metadynamics. *Journal of Physical Chemistry B* **110**, 3533–3539 (2006).

[25] Bussi, G. & Laio, A. Using metadynamics to explore complex free-energy landscapes. *Nature Reviews Physics* **2**, 200–212 (2020).

[26] Branduardi, D., Gervasio, F. L. & Parrinello, M. From a to b in free energy space. *The Journal of Chemical Physics* **126**, 054103 (2007).

[27] McGibbon, R. T. *et al.* Mdtraj: A modern open library for the analysis of molecular dynamics trajectories. *Biophysical Journal* **109**, 1528 – 1532 (2015).

[28] Scherer, M. K. *et al.* PyEMMA 2: A Software Package for Estimation, Validation, and Analysis of Markov Models. *Journal of Chemical Theory and Computation* **11**, 5525–5542 (2015).

[29] Pedregosa, F. *et al.* Scikit-learn: Machine learning in Python. *Journal of Machine Learning Research* **12**, 2825–2830 (2011).

[30] Balevičius, V. *et al.* Fine control of chlorophyll-carotenoid interactions defines the functionality of light-harvesting proteins in plants. *Scientific Reports* **7**, 1–10 (2017).

[31] Fox, K. F. *et al.* A possible molecular basis for photoprotection in the minor antenna proteins of plants. *Biochimica et Biophysica Acta - Bioenergetics* **1859**, 471–481 (2018).

[32] Daskalakis, V. *et al.* Structural basis for allosteric regulation in the major antenna trimer of photosystem ii. *Journal of Physical Chemistry B* **123**, 9609–9615 (2019).

[33] Madjet, M. E., Abdurahman, A. & Renger, T. Intermolecular Coulomb Couplings from Ab Initio Electrostatic Potentials: Application to Optical Transitions of Strongly Coupled Pigments in Photosynthetic Antennae and Reaction Centers. *Journal of Physical Chemistry B* **110**, 17268–17281 (2006).

[34] Andreussi, O., Knecht, S., Marian, C. M., Kongsted, J. & Mennucci, B. Carotenoids and light-harvesting: From dft/mrci to the tamm-dancoff approximation. *Journal of Chemical Theory and Computation* **11**, 655–666 (2015).

[35] Nottoli, M. *et al.* The role of charge-transfer states in the spectral tuning of antenna complexes of purple bacteria. *Photosynthesis research* **137**, 215–226 (2018).

[36] Curutchet, C., Scholes, G. D., Mennucci, B. & Cammi, R. How solvent controls electronic energy transfer and light harvesting: toward a quantum-mechanical description of reaction field and screening effects. *The Journal of Physical Chemistry B* **111**, 13253–13265 (2007).

[37] Cupellini, L., Jurinovich, S., Prandi, I. G., Caprasecca, S. & Mennucci, B. Photoprotection and triplet energy transfer in higher plants: the role of electronic and nuclear fluctuations. *Phys. Chem. Chem. Phys.* **18**, 11288–11296 (2016).

[38] You, Z.-Q. & Hsu, C.-P. Theory and calculation for the electronic coupling in excitation energy transfer. *Int. J. Quantum Chem.* **114**, 102–115 (2014).

[39] Cupellini, L., Calvani, D., Jacquemin, D. & Mennucci, B. Charge transfer from the carotenoid can quench chlorophyll excitation in antenna complexes of plants. *Nature Communications* **11**, 662 (2020).

[40] Ceriotti, M., Tribello, G. A. & Parrinello, M. Simplifying the representation of complex free-energy landscapes using sketch-map. *Proceedings of the National Academy of Sciences* **108**, 13023–13028 (2011).

[41] Frisch, M. J. *et al.* Gaussian~16 Revision A.03 (2016). Gaussian Inc. Wallingford CT.

[42] Bondanza, M., Jacquemin, D. & Mennucci, B. Excited states of xanthophylls revisited: Toward the simulation of biologically relevant systems. *The Journal of Physical Chemistry Letters* **12**, 6604–6612 (2021).

[43] Silva-Junior, M. R. & Thiel, W. Benchmark of electronically excited states for semiempirical methods: Mndo, am1, pm3, om1, om2, om3, indo/s, and indo/s2. *Journal of chemical theory and computation* **6**, 1546–1564 (2010).

[44] Dral, P. O. *et al.* Semiempirical quantum-chemical orthogonalization-corrected methods: theory, implementation, and parameters. *Journal of chemical theory and computation* **12**, 1082–1096 (2016).

[45] Keal, T. W., Wanko, M. & Thiel, W. Assessment of semiempirical methods for the photoisomerisation of a protonated schiff base. *Theoretical Chemistry Accounts* **123**, 145–156 (2009).
