# Supplementary material for: A different perspective for nonphotochemical quenching in plant antenna complexes
Source: Nat Commun. 2021 Dec 9;12:7152. doi: 10.1038/s41467-021-27526-8 (PMC8660843; doi:10.1038/s41467-021-27526-8)
Supplement: Supplementary file 3 — Reporting Summary [file 41467_2021_27526_MOESM3_ESM.pdf]

nature portfolio

Corresponding author(s): Lorenzo Cupellini
Francesco Luigi Gervasio
Benedetta Mennucci

Last updated by author(s): Nov 15, 2021

Reporting Summary

Nature Portfolio wishes to improve the reproducibility of the work that we publish. This form provides structure for consistency and transparency in reporting. For further information on Nature Portfolio policies, see our Editorial Policies and the Editorial Policy Checklist.

Statistics

For all statistical analyses, confirm that the following items are present in the figure legend, table legend, main text, or Methods section.

n/a	Confirmed	
☐	☒	The exact sample size (*n*) for each experimental group/condition, given as a discrete number and unit of measurement
☐	☒	A statement on whether measurements were taken from distinct samples or whether the same sample was measured repeatedly
☒	☐	The statistical test(s) used AND whether they are one- or two-sided<br>*Only common tests should be described solely by name; describe more complex techniques in the Methods section.*
☒	☐	A description of all covariates tested
☒	☐	A description of any assumptions or corrections, such as tests of normality and adjustment for multiple comparisons
☐	☒	A full description of the statistical parameters including central tendency (e.g. means) or other basic estimates (e.g. regression coefficient) AND variation (e.g. standard deviation) or associated estimates of uncertainty (e.g. confidence intervals)
☒	☐	For null hypothesis testing, the test statistic (e.g. *F*, *t*, *r*) with confidence intervals, effect sizes, degrees of freedom and *P* value noted<br>*Give P values as exact values whenever suitable.*
☒	☐	For Bayesian analysis, information on the choice of priors and Markov chain Monte Carlo settings
☒	☐	For hierarchical and complex designs, identification of the appropriate level for tests and full reporting of outcomes
☒	☐	Estimates of effect sizes (e.g. Cohen's *d*, Pearson's *r*), indicating how they were calculated

Our web collection on statistics for biologists contains articles on many of the points above.

Software and code

Policy information about availability of computer code

Data collection	Amber 18, https://ambermd.org/<br>Gromacs 2018.6, https://www.gromacs.org/<br>PLumed 2.5.1, https://www.plumed.org/<br>Gaussian 16, https://gaussian.com/<br>MNDO 2020, https://www.kofo.mpg.de/en/institute/history/1993-to-present/theoretical-chemistry
Data analysis	MDTraj v 1.9.5<br>PyTraj v 2.0.5<br>Scipy v 1.6.0<br>NumPy v 1.20.1<br>Scikit-Learn v 0.24.1<br>Matplotlib v 3.3.4<br>Seaborn v 0.11.1<br>PyEMMA v 2.5.7<br>ParmED v 3.0.0<br>CPPTRAJ v 18.01,<br>VMD v 1.9.4a37<br>UCSF Chimera v 1.15

For manuscripts utilizing custom algorithms or software that are central to the research but not yet described in published literature, software must be made available to editors and reviewers. We strongly encourage code deposition in a community repository (e.g. GitHub). See the Nature Portfolio guidelines for submitting code & software for further information.

Data

Policy information about availability of data

All manuscripts must include a data availability statement. This statement should provide the following information, where applicable:

- Accession codes, unique identifiers, or web links for publicly available datasets
- A description of any restrictions on data availability
- For clinical datasets or third party data, please ensure that the statement adheres to our policy

The datasets generated during and/or analysed during the current study are available in the Zenodo repository, https://zenodo.org/record/5703113.

Field-specific reporting

Please select the one below that is the best fit for your research. If you are not sure, read the appropriate sections before making your selection.

☒ Life sciences ☐ Behavioural & social sciences ☐ Ecological, evolutionary & environmental sciences

For a reference copy of the document with all sections, see nature.com/documents/nr-reporting-summary-flat.pdf

Life sciences study design

All studies must disclose on these points even when the disclosure is negative.

Sample size	Extension of the each simulation was decided on the basis of simulation convergence, compatibly with the available computational resources. Sample sizes for quantum chemical calculations were decided based on the available computational resources and sample representativity of the simulation.<br>Statistical algoritmhs such as farthest point sampling were employed to select the samples prior to quantum chemical calculations.
Data exclusions	Some data coming from the parallel tempering in the well-tempered ensemble simulation has been excluded on the basis of its statistical weight. Samples with a low statistical weight have been excluded from the analysis, as explained in the Supplementary Information file. Approximately 50% of the simulation data has been retained for the analysis as a result of filtering out samples with low weight. For the unbiased molecular dynamics trajectories, only the "production" part of the trajectory is reported, i.e., minimization, heating, and equlibration are excluded from the analysis.
Replication	For the parallel tempering in the well-tempered ensemble simulation, one simulation of 15 replicas in parallel has been run and analyzed.<br>For the unbiased molecular dynamics trajectories of CP29, referred in the manuscript as cMD_CryoEM, one replica has been run and analyzed.<br>For the unbiased simulations starting from the Open basin, referred in the manuscript as cMD_Open, two replicas have been run and analyzed.<br>For the metadynamics simulations connecting CP29_CryoEM to CP29_Open, two replicas, one starting from the CryoEM basin and one from the Open basin, have been run and analyzed.
Randomization	We do not carry out any randomizations in our analysis.
Blinding	We do not carry out any blinding in our data collection and analysis.

Reporting for specific materials, systems and methods

We require information from authors about some types of materials, experimental systems and methods used in many studies. Here, indicate whether each material, system or method listed is relevant to your study. If you are not sure if a list item applies to your research, read the appropriate section before selecting a response.

Materials & experimental systems

n/a	Involved in the study	
☒	☐	Antibodies
☒	☐	Eukaryotic cell lines
☒	☐	Palaeontology and archaeology
☒	☐	Animals and other organisms
☒	☐	Human research participants
☒	☐	Clinical data
☒	☐	Dual use research of concern

Methods

n/a	Involved in the study	
☒	☐	ChIP-seq
☒	☐	Flow cytometry
☒	☐	MRI-based neuroimaging
